# Sisters Acts: Converging Signaling Between CaMKII and CaMKIV, Two Members of the Same Family

**Published:** 2012-10-11

**Authors:** M.R. Rusciano, A.S. Maione, M. Illario

**Affiliations:** Department of Cellular and Molecular Biology and Pathology, Federico II University, Italy

**Keywords:** calcium, cell signaling, kinase, proliferation

## Abstract

Calcium (Ca^2+^) is a universal second messenger that regulates a number of diverse cellular processes including cell proliferation, development, motility, secretion, learning and memory^1^, ^2^. A variety of stimuli, such as hormones, growth factors, cytokines, and neurotransmitters induce changes in the intracellular levels of Ca^2+^. The most ubiquitous and abundant protein that serves as a receptor to sense changes in Ca^2+^ concentrations is Calmodulin (CaM), thus mediating the role as second messenger of this ion. The Ca^2+^/CaM complex initiates a plethora of signaling cascades that culminate in alteration of cell functions. Among the many Ca^2+^/CaM binding proteins, the multifunctional protein kinases CaMKII and CaMKIV play pivotal roles in the cell.

## I. INTRODUCTION

The general structure of CaMKs includes an N-terminal kinase domain, an autoregulatory domain, an overlapping CaM-binding domain and, in phosphorylase kinase and CaMKII, a C-terminal association domain that is essential for multimerization and targeting.

The best characterized CaM Kinase is CaMKII^3^. CaMKII is a multimeric enzyme composed of 12 subunits and it is encoded by 4 separate genes (α, β, γ, δ) with at least 24 peptides generated by alternate splicing^4^, ^5^ and at least one isoform expressed in every cell type^6^. CaMKII has a distinct mechanism of regulation that differs from the others CaM kinases. One catalytic subunit phosphorylates the autoinhibitory domain of the adjacent subunit on T286 (in the α isoform). This event requires that both the catalytic subunit and the substrate subunit are bound to Ca^2+^/CaM^7^, ^8^. T286 phosphorylation then results in 20–80% Ca^2+^/CaM-independent activity^4^,^9^–^13^. Autophosphorylation of T286 increases affinity for CaM by decreasing the rate of CaM dissociation. CaM is trapped by autophosphorylation, so that even when Ca^2+^ levels are reduced, the kinase is fully active until CaM dissociates (several hundreds of seconds^13^). This could serve as a mechanism to increase the sensitivity of CaMKII to the changes in intracellular Ca^2+^ concentration^7^, ^13^.

**Figure:**
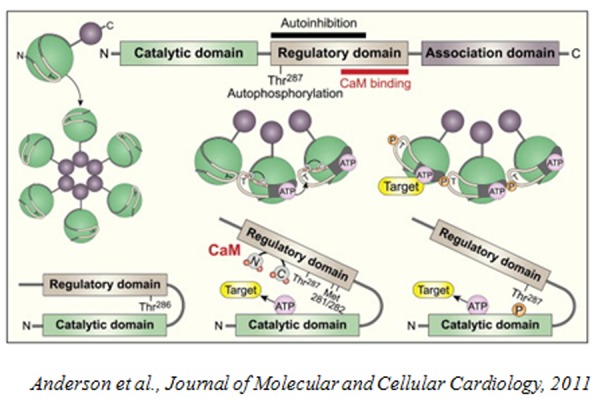
Anderson et al., Journal of Molecular and Cellular Cardiology, 2011

CaMKIV is a serine/threonine protein kinase that has been localized also in the nucleus^14^. Its expression is tissue-specific, with expression restricted primarily to distinct regions of the brain, T-lymphocytes, and postmeiotic germ cells,^15^, ^16^ although it has been found in other cell types 17, being especially enriched in cerebellar granule cells. CaMKIV (one gene, two splice variants)^18^ – is a monomeric enzyme, and apart from activation by Ca^2+^/CaM, shows very different modes of regulation by phosphorylation compared to CaMKII. CaMKIV has an “activation loop” phosphorylation site that is absent in CaMKII. Binding of Ca2+/CaM to CaMKIV exposes this activation loop site to allow phosphorylation by the upstream CaMKK, when it is simultaneously activated by Ca^2+^/CaM^19^. Phosphorylation of the activation loop in CaMKIV primarily increases its Ca^2+^/CaM-dependent activities.

**Figure:**
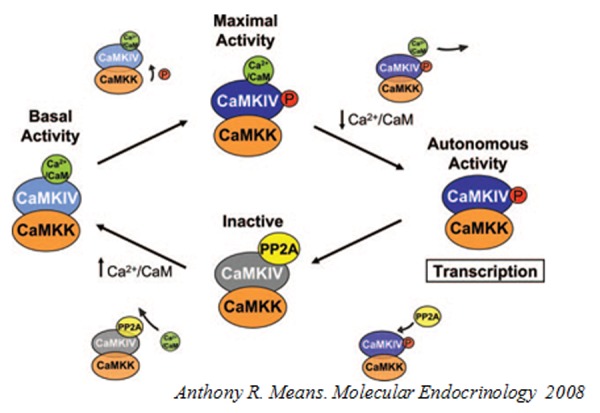
Anthony R. Means. Molecular Endocrinology 2008

## II. CaMK-MEDIATED ACTIVATION OF TRANSCRIPTION

### CaMKII and CREB

As CaM kinases II and IV have quite similar substrate specificity determinants, it is not completely surprising that they sometimes phosphorylate the same proteins. One such in vitro substrate for these kinases is the cAMP-response element binding protein, CREB. CaMKII can phosphorylate CREB at Ser133 residue leading to the speculation that CaMKII mediates the Ca^2+^ requirement for expression of the immediate early genes^5^. However, while the truncated form of CaMKII can stimulate CREB-mediated transcription in some cells, it is inhibitory in others. Sun et al. 20 discovered that in addition to Ser-133, CaMKII also phosphorylated a second residue on CREB, Ser-142. Indeed, phosphorylation of Ser-142 was not only inhibitory, but this modification was also dominant and could reverse the activation of CREB resulting from its phosphorylation on Ser-133 by PKA. This phosphorylation seems to be destabilizing for the association between CREB and CBP^21^. Interestingly, the nature of the effect of CaMKII on transcription is both cell and promoter dependent.

### CaMKIV AND CREB

CaMKIV shows very strong nuclear localization^22^, ^23^, and many studies support the idea that it is responsible for Ca^2+^-dependent stimulation of transcription through phosphorylation of CREB and serum response factor (SRF)^5^, ^22^, ^24^. Activation by CaMKIV occurs via direct phosphorylation of the activating serines of these transcription factors, Ser133 (CREB), Ser63 (ATF-1), and Ser103(SRF), respectively^25^. CaMKIV phosphorylates CREB Ser133, the same site that is phosphorylated by PKA. Transfected CaMKIV alone is a relatively poor stimulator of transcriptional activation by CREB: indeed, cotransfection of CaMKK with CaMKIV gives a 14-fold enhancement of transcription^26^. Studies in cultured hippocampal neurons indicate that CaMKIV regulates CREB-dependent gene transcription in response to electrical stimulation or KCl depolarization^27^. This role of CaMKIV in CREB-mediated transcription has been confirmed in transgenic mice that express an inactive form of CaMKIV only in T cells in the thymus^27^. Overexpression of inactive CaMKIV would be expected to function in a dominant negative manner. These thymic T cells have a reduced ability, upon stimulation, to phosphorylate CREB, induce transcription of FosB and produce interleukin 2 (IL-2)^28^. There is also good evidence for involvement of CaMKIV in transcriptional regulation of the BDNF gene through phosphorylation of a CREB family member^29^.

These observations provide a mechanism that would permit the Ca^2+^ signaling pathway to be either antagonistic or additive with the cAMP pathway for activation of CREB, depending on the relative activity of specific CaM kinases.

## III. CaMKs MEDIATED REGULATION OF APOPTOSIS

Bok et al^30^ observed that CaMKII promotes SGN survival, at least in part, by functionally inactivating Bad. The ability of Bad to move from the cytoplasm to the mitochondria, where it can carry out its pro-apoptotic function, is regulated by phosphorylation 31, 32. Thus, Bad plays a central role in the regulation of apoptosis. CaMKII also regulates apoptosis by inactivating Bad. One phosphorylation site on Bad, Ser170^33^, is a potential CaMKII target, raising the possibility that CaMKII phosphorylates Bad directly. However, co-expression of Bad and truncated form of CaMKII(1–290) in PC12 cells results in Bad hyper-phosphorylation, including phosphorylation of Ser112. This implies an indirect pathway for Bad phosphorylation by CaMKII. The mechanism by which CaMKII inactivates Bad involves multiple signaling pathways, and differs among cell types. CaMKII also suppresses nuclear translocation of histone deacetylase, thereby promoting neuronal survival^34^. Indeed, CaMKII has been shown to activate the pro-survival transcriptional regulator NF-κB in T lymphocytes and in neurons^35^. Because dominant-negative CREB constructs do not reduce the pro-survival effect of CaMKII, it is unlikely that CREB is the nuclear target of CaMKII. The depolarization also promotes survival by recruiting a nuclear pathway involving CaMKIV and CREB^30^. This is supported by the observations that dominant-inhibitory CaMKIV and dominant-inhibitory CREB both reduce the ability of depolarization to promote survival and dominant-inhibitory CREB blocks the ability of CaMKIV to promote survival. They also used a constitutively-active CREB mutant, CREBDIEDML, and found that it failed to support SGN survival. Probably the level of transcriptional activation given by CREBDIEDML is insufficient to promote survival. Alternatively, recruitment of CBP by CREB is necessary but is not sufficient for promotion of survival via CREB-dependent gene expression.

## IV. CaMKs MEDIATED REGULATION OF PROLIFERATION

Cell proliferation is regulated by converging signals on the cell cycle machinery that determine whether the cell stays in the G_1_ phase or proceeds to S phase. The progression through G_1_ into the DNA synthesizing S phase is driven by cyclin-dependent kinase (CDK)4 and CDK6, that interact with the cyclin D family of proteins, and CDK2, that interacts with cyclins A/E 36. The Ras/Raf/Mek/Erk cascade plays a pivotal role in the control of this process: indeed, sustained Erk activation is required to pass the G_1_ restriction point and regulate cyclin D1 expression during mid-G1 phase 37, 38. CaMKII plays a pivotal role in the modulation of Erk activation in a number of cell models. A crosstalk between CaMKII and Erk pathway was first demonstrated in response to cell adhesion to the extracellular matrix in thyroid cells. CaMKII participates to Raf1 activation and controls Erk phosphorylation following integrin stimulation by fibronectin 39, 40. Indeed, the link between Ca^2+^ signaling and the ERK pathway has been documented 38, 41: ERK is activated by a CaMKII and Raf-dependent mechanism 42, and CaMKII facilitates adhesion-dependent activation of ERK in VSMCs 41, 43. CaM antagonist or CaMKII inhibitors attenuate ERK activation in response to several stimuli 44, and coexpression of CaMKII or a CaMKII inactive mutant in CHO cells down-regulates Ca^2+^-induced ERK activation 15, 45. These data suggest that CaMKII and ERK are essential mediators of cell proliferation 46, 47. The role of CaMKII in cell proliferation is not a restricted mechanism, but it is a general phenomenon that may be relevant for the biological effects of many growth factors and hormones.

## V. CaMKs MEDIATED REGULATION OF DIFFERENTIATED FUNCTIONS

### SURVIVAL

The multifunctional CaMKs family proteins are involved in the control of differentiation and survival of neurons and hematopoietic stem cells 48. In the cerebellum, granule and Purkinje cells (PCs) develop synergistically, and alterations in the developmental program of either cell type affects the other 45. Many studies showed that the absence of CaMKIV results in abnormal PCs, characterized by a decreased number of mature cells together with stunted arborization and altered parallel fiber synaptic currents of the remaining cells 21, 49. Kobubo et al hypothesized that these adult defects may arise from developmental issues involving CGCs in addition to PCs. These cells only express CaMKIV during a briefperiod between late embryogenesis and early postnatal development, whereas CGCs express both CaMKIV and its upstream activator CaMKK2 from early postnatal development through adulthood 50 .CaMKIV exert prosurvival functions. Inneurons, BDNF signaling through TrKB inhibits apoptosis through the MAP and PI-3 kinase/AKT pathways 15. CaMKIV has a prosurvival role in multiple cell types including hematopoietic stem cells(HSCs) 51, and dendritic cells 52.

#### Kitsos

The hematopoietic stem cell (HSC) gives rise to all mature, terminally differentiated cells of the blood. CaMKIV is involved in early hematopoietic development, and the absence of CaMKIV results in a reduction in the number of c-Kit^+^ScaI^+^Lin^−/low^ cells (KLScells), a cell population that includes long-term and short term hematopoietic stem cells as well as other multipotent progenitor cells 53. Camk4 gene is expressed in KLS cells, and CaMKIV is required for KLS cells to repopulate the bone marrow in transplantation assays. Camk4^−/−^KLS cells display enhanced proliferation as well as increased apoptosis, in vivo and in vitro, compared with wild type (WT) cells and have decreased levels of phospho-CREB (pCREB), CBP, Bcl-2 mRNA and Bcl-2 protein. Re-expression of CaMKIV in Camk4^−/−^KLS cells restores Bcl-2 and CBP levels and rescues the proliferation defects.

Many critical biological functions involve Ca^2+^ signaling in DC. For example, apoptotic body engulfment and processing are accompanied by a rise in intracellular Ca^2+^ and are dependent on external Ca^2+^ 54. In addition, chemotactic molecules produce Ca^2+^ increases in DC, 55 suggesting the involvement of a Ca^2+^-dependent pathway in the regulation of DC migration. The role of a Ca^2+^-dependent pathway in the mechanism regulating DC maturation is suggested by the opposite effects induced by Ca^2+^ ionophores or chelation of extracellular Ca^2+^ on this process^56^. The pharmacologic inhibition of CaMKs as well as ectopic expression of kinase-inactive CaMKIV decrease the viability of monocyte-derived DCs exposed to bacterial LPS. Although isolated *Camk4* / DCs are able to acquire the phenotype typical of mature cells and release normal amounts of cytokines in response to LPS, they fail to accumulate pCREB, Bcl-2, and Bcl-xL and therefore do not survive.

### CARDIAC HYPERTROPHY

CaMKII has been implicated in several key aspects of acute cellular Ca^2+^ regulation related to cardiac excitation-contraction (E-C) coupling. CaMKII phosphorylates sarcoplasmic reticulum^57^ proteins, including the ryanodine receptors (RyR2) and phospholamban (PLB)^57^. Contractile dysfunction develops with hypertrophy, characterizes heart failure, and is associated with changes in cardiomyocyte Ca^2+^ homeostasis 58. CaMKII expression and activity are altered in the myocardium of rat models of hypertensive cardiac hypertrophy^59^ and heart failure 60, and in cardiac tissue from patients with dilated cardiomyopathy^61^. Several transgenic mouse models have confirmed a role for CaMK in the development of cardiac hypertrophy. Hypertrophy develops in transgenic mice that overexpress CaMKIV 62, but this isoform is not detectable in the heart and CaMKIV knockout mice still develop hypertrophy following transverse aortic constriction (TAC) 63. CaMKII regulates expression of several hypertrophic marker genes, including ANF^64^ BNP^65^, h-MHC^66^ and a-skeletal actin^61^. The nuclear localization signal of CaMKIIδB was shown to be required for this hypertrophic response, as transfection of CaMKIIδC did not result in enhanced ANF expression^67^, ^68^. MEF2 has been suggested to act as a common endpoint for hypertrophic signaling pathways in the myocardium,^66^ and studies using CaMKIV transgenic mice crossed with MEF2 indicator mice suggest that MEF2 is a downstream target for CaMKIV 69. Recent studies have demonstrated that MEF2 can interact with class II histone deacetylases (HDACs), a family of transcriptional repressors, as well as with other repressors that limit MEF2-dependent gene expression. Notably, constitutively activated CaMKIV have been shown to activate MEF2 by phosphorylating and dissociating HDACs, leading to its subsequent nuclear export 70.

## VI. CaMKs AND INFLAMMATION

Sepsis is a special type of host inflammatory response to bacterial infection that originates from massive and widespread release of pro-inflammatory mediators. Bacterial endotoxins, such as LPS, are the major offending factors in sepsis that activate TLR-mediated signaling to generate inflammatory response that is amplified in a self-sustaining manner. There are meny evidences of a correlation between multifunctional CaM kinases and TLR-4 signaling. CaMKII directly phosphorylates components of TLR signaling, and promotes cytokine production in macrophages^71^. Complement activation is also a recognized factor in the pathogenesis of sepsis. Inhibition of the complement cascade decreases inflammation and improves mortality in animal models^51^. Differentiation and survival of antigen presenting dendritic cells (DC) uponTLR-4 activation requires CaMKIV^72^. DC from CaMKIV−/−mice failed to survive upon LPS-mediated TLR-4 induction. However, ectopic expression of CaMKIV was able to rescue this defect. In another study, the selective inhibition of CaMKII interfered with terminal differentiation of monocyte-derived DCs by preventing up-regulation of co-stimulatory and MHC II molecules as well as secretion of cytokines induced by TLR-4 agonists^73^. Thus, CaM kinases seem to play a general role in inflammatory processes

## VII. CONCLUSIONS

CaMKs define a family of ser-thr kinases that direct a wide range of cellular processes and cell fate decisions. Since their discovery, much of the focus has been on their regulation of memory and learning. In recent years, studies on CaMKII and CaMKIV signaling in a number of cell models have established the importance of the Ca^2+^-CaM-CaMKK-CaMKs pathways in effecting proliferation, survival, differentiation and associated molecular events. Intriguing new findings also indicate that, although the two kinases might share some substrates, there is specificity in the pathways they contribute, thus reflecting both shared and unique properties. The emergence of ERK as a critical CaMKII regulatory target for cell proliferation has united membrane proximal regulatory events orchestrated by the Ras activated cascade with key transcriptional CaMKs targets.

Ca^2+^ is ubiquitously present in the cells, hence its compartimentalization and the regulation of its downstream kinases need to be finely tuned, in order to efficiently regulate biological functions. The involvement of CaMKII and CaMKIV in pathways that regulate functions as different as proliferation, survival and differentiation imply numerous cross-talks and their harmonization. Both kinases require Ca^2+^ increases to be activated, although other events are required to support their differential activation. Subcellular compartimentalization provides another tool to distinctively activate CaMKII and CaMKIV depending upon the cell’s needs. It is possible, though, to hypothesize a further mechanism of counter-regulation between the two kinases: insights into the regulation and impact of a crosstalk between CaMKII and CaMKIV signaling might bring in new highlights for biological functions, and their disruption in human diseases.
